# Editorial: Impact of family health and habits on children's oral health

**DOI:** 10.3389/froh.2025.1689517

**Published:** 2025-09-11

**Authors:** Ziad D. Baghdadi

**Affiliations:** Department of Preventive Dental Sciences and Centre for Community Oral Health, University of Manitoba, Winnipeg, MB, Canada

**Keywords:** maternal health, maternal habits, child's oral health, maternal nutrition, maternal oral hygiene, preventive dentistry

A complex interplay of biological, behavioral, and environmental factors shapes the oral health of children. At the center of this nexus lies the family—a child's first source of care, habits, and health behaviors. This Research Topic, Impact of Family Health and Habits on Children's Oral Health, brings together diverse investigations that collectively underscore the central role of the family unit in either mitigating or exacerbating oral health risks in childhood.

This collection provides new insights into how systemic health conditions, family routines, parental knowledge, and broader community-level policies intersect to influence pediatric oral health outcomes. The findings highlight the imperative of holistic, family-centered approaches in both clinical care and public health planning.

The link between systemic health and oral health is explored in the study “Impact of Severity of Bronchial Asthma on Oral Health in Children” by Alrashdi and Alyhya. Here, the authors demonstrate that children with severe asthma experience higher rates of dental caries and gingival inflammation, possibly due to medication side effects, altered salivary flow, or parental preoccupation with respiratory management at the expense of oral hygiene. This article reminds us that chronic illness within the family often redistributes caregiving attention in ways that can unintentionally neglect oral care. Additionally, addressing modifiable risk factors may be beneficial in enhancing oral health in children with asthma.

From an environmental and public health perspective, the article “Associations between community water fluoridation cessation and the prevalence of dental caries and fluorosis in Alrass city, Saudi Arabia” by Alrashdi reveals the tangible consequences of health policy decisions on children's oral health. The cessation of fluoridation in Alrass was associated with an increased prevalence of dental caries, reaffirming the protective role of fluoridated water and the importance of maintaining preventive public health interventions, especially in communities with limited access to dental care.

On a more intimate level, “Family factors associated with dental caries among 5-year-old preschool children” by Castilho et al. explores socioeconomic and behavioral influences. The study found that lower parental education and income, irregular dental visits, and diets high in sugar were significantly associated with an increased prevalence of caries in young children. This finding reinforces the interdependence of family structure, parental awareness, and socioeconomic conditions in shaping oral health trajectories from an early age. The attitudes of caregivers regarding oral health have a considerable influence on the dental health of their children.

Building on this theme, “May family routines impact oral health in American children?” by Pardi et al. Examines the subtle daily habits that contribute to oral health disparities. The study reveals that consistent family routines around mealtimes, bedtimes, and toothbrushing practices are associated with lower rates of dental disease. This article supports the emerging narrative that stable household routines—often overlooked in clinical settings—can be powerful predictors of oral health outcomes.

The article “Parental awareness and dental health behavior of children with congenital heart disease, with diabetes mellitus, or undergoing anti-cancer treatment, compared to healthy children” by Halperson et al. presents a compelling comparison across vulnerable pediatric populations. The authors highlight significant gaps in parental awareness and preventive dental practices among medically compromised children. The findings underscore the importance of integrating medical and dental education for caregivers, particularly for families managing complex health conditions where oral health may be overlooked. It highlights the importance of clear and continuous communication between pediatricians and dentists.

Finally, “Unusual oral manifestation of Kindler syndrome: a case report and review of literature” by Bhandary et al. reminds us of the diagnostic and care complexities in rare genetic disorders. While this case may appear isolated, it highlights the crucial role of family observation and engagement in identifying early oral manifestations of systemic conditions, particularly in underserved or medically vulnerable populations.

In sum, this Research Topic reinforces that children's oral health cannot be separated from the broader ecosystem of family health, knowledge, and habits. Each article contributes a unique piece to a larger mosaic that demonstrates the family's role not just as a context, but as a determinant of pediatric oral health. For clinicians, researchers, and policymakers, these findings make a compelling case for integrated, family-centered models of oral health promotion that extend beyond the dental chair and into the heart of the household.

We hope this collection inspires continued interdisciplinary dialogue and innovation, aimed at empowering families, strengthening policies, and ultimately securing healthier futures for children worldwide.

